# Buffalo Medical and Surgical Association

**Published:** 1891-10

**Authors:** W. S. Renner

**Affiliations:** Secretary


### BUFFALO MEDICAL AND SURGICAL ASSOCIATION.

Reported by W. S. RENNER, M. D., Secretary.

*Stated Meeting, August 4th.*

The President being absent, Dr. Bartlett occupied the chair.

Dr. Irving M. Snow read a paper, entitled, Lavage and Intestinal Irrigation in the Digestive Disorders of Infants.

### DISCUSSION.

Dr. Coakley does not think that the cause of the digestive disorders is always in the alimentary canal. He thinks the cause is often to be found in the state of adjacent organs. In hot climates he thinks the diarrhea, cholera infantum, and dysentery are due to the great heat. He thinks that the proper treatment is to increase the intestinal secretions, and that Dr. Snow claims too much for lavage.

Dr. Thoma related the case of a child with acute mycotic diarrhea which he thought might have been saved had he resorted to intestinal irrigation.

Dr. Van Peyma thought that heat affected the quality of the food ingested.

Dr. Congdon thinks that children do not require as much food in warm weather; that nature tries to free the child of the excess; and that the secretions are not supplied in abundance in hot weather. He thinks that lavage is an easy method of freeing the stomach of an excess of food.

Dr. Persons said that lavage had given him better satisfaction than any other method of treatment. It will not cure all conditions; chronic cases require more. Intestinal irrigation has also given him great satisfaction.

In treating these digestive disorders, the indications are: First, elimination; second, dietetic treatment; third, treatment of symptoms; fourth, treatment of any constitutional condition which may exist. We often do not take into consideration influences brought to bear by external circumstances. He uses antiseptics, artificial foods, and opiates when indicated.

Dr. Goltra asked if it were well, in intensely acute conditions, to wash out the stomach and intestines. He thought that then we must take into consideration the impaired condition of the blood. He believes in the use of partially digested foods.

Dr. B. G. Long thinks that by both these methods water is supplied to the system in the very acute cases. He has great faith in the use of sterilized milk.

Dr. Hayd said that the longer he employed lavage the better he liked it, and that the longer he practised medicine the less he depended upon drugs in the treatment of these conditions. Many attacks of diarrhea in children can be prevented by wearing an abdominal woolen bandage. He has not used lavage much in acute conditions; depends more on nature. He thinks the best thing for children with acute intestinal trouble is to prevent them from taking food; give them some thin gruel, barley water, or toast water.

The remedy in chronic gastritis is lavage; it carries off mucus, stimulates the stomach walls, and furthers the supply of gastric juice.

Dr. Bartlett said that he was one of the first to use injections per rectum of bichloride of mercury solution in dysentery.

For children he prefers small doses of iodide of mercury to any other treatment. Two things he had found of importance in treating diarrhea of children. First, to take the temperature per rectum, to find out the amount of local disturbance; and, secondly, to give bromidia to quiet the child. He has never been favorably impressed by the use of prepared foods. The chief thing for the child is to get it into the country.

Dr. Snow, in reply, said he had never had any difficulty in getting parents to consent to have their child's stomach washed out. In reply to Dr. Goltra, he said that he used opiates in cholera infantum. In chronic indigestion, lavage increases the amount of hydrochloric acid, and you have added to the gastric juice what is needed without giving partially digested foods.

In reply to Dr. Long, he stated that he had mentioned that water is taken up by the stomach and intestines during the use of these methods. He held that sterilized water acted fully as well as a solution of bichloride of mercury.

Dr. Bartlett said that it had become the sad duty of the Association to take action on the death of Dr. Frank Hamilton Potter, a member of this Association, and at one time its secretary. He appointed Drs. Persons, Coakley, and B. G. Long a committee to draft resolutions. The committee presented the following

### MEMORIAL.

Whereas, This Association has lost one of its most valued members in the death of its former secretary, Dr. Frank H. Potter, whose earnest zeal and untiring energy have done much to enrich the records of this Association; whose civil and professional achievements are justly recognized by his colleagues; and whose social and private life affords a noble example for emulation; therefore,

*Resolved,* That the Buffalo Medical and Surgical Association desires to perpetuate his memory, tenders to his bereaved family an expression of its sympathy in their loss, and directs that this preamble and resolution be spread upon its minutes.

ALBERT E. PERSONS,
J. B. COAKLEY,
B. G. LONG,
*Committee.*

On motion of Dr. Brecht, seconded by Dr. Hayd, the memorial was adopted as read, and it was directed that a copy of the memorial be given the family, and that it likewise be entered upon the records of this Association.

